# Deciphering Structural and Dynamical Properties of Hydrated Cobalt Porphyrins via Ab Initio Quantum Mechanical Charge Field Molecular Dynamics Simulation

**DOI:** 10.1021/acs.jpcb.3c00837

**Published:** 2023-05-23

**Authors:** Sehrish Jamal, Zobia Naz, Syed Tarique Moin, Thomas S. Hofer

**Affiliations:** Third World Center for Science and Technology, H.E.J. Research Institute of Chemistry International Center for Chemical and Biological Sciences, University of Karachi, Karachi-75270, Pakistan; Theoretical Chemistry Division, Institute of General, Inorganic and Theoretical Chemistry, University of Innsbruck, Innrain 80-82, A-6020 Innsbruck, Austria

## Abstract

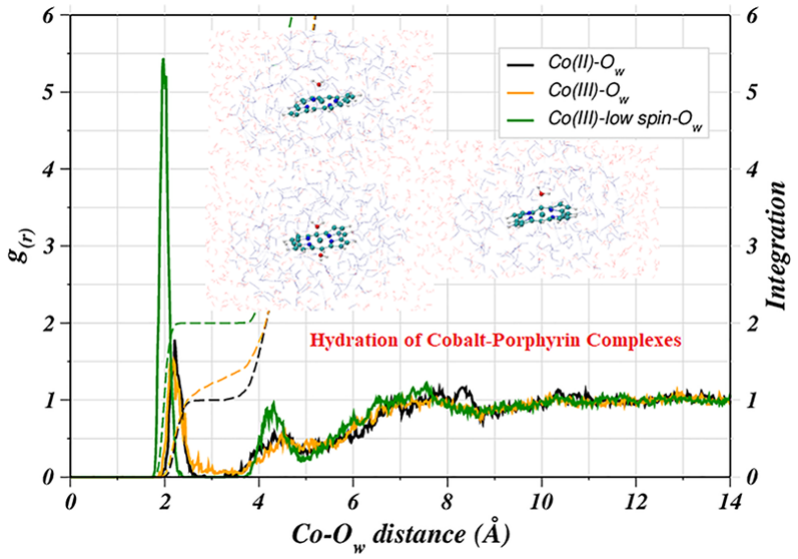

The present study successfully implemented the ab initio quantum mechanical charge field molecular dynamics (QMCF MD) formalism for the investigation of structural and dynamical properties of hydrated cobalt–porphyrin complexes. Considering the significance of cobalt ions in biological systems (for instance, vitamin B12), which reportedly incorporate cobalt ions in a d6, low spin, +3 state chelated in the corrin ring, an analog of porphyrin, the current study is focused on cobalt in the oxidation states +2 and +3 bound to the parent porphyrin lead structures embedded in an aqueous solution. These cobalt–porphyrin complexes were investigated in terms of their structural and dynamical properties at the quantum chemical level. The structural attributes of these hydrated complexes revealed the contrasting features of the water binding to these solutes, including a detailed evaluation of the associated dynamics. The study also yielded notable findings in regard to the respective electronic configurations vs coordination, which suggested that Co(II)-POR possesses a 5-fold square pyramidal coordination geometry in an aqueous solution containing the metal ion coordinating to four nitrogen atoms of the porphyrin ring and one axial water as the fifth ligand. On the other hand, high-spin Co(III)-POR was hypothesized to be more stable due to the smaller size-to-charge ratio of the cobalt ion, but the high-spin complex demonstrated unstable structural and dynamical behavior. However, the corresponding properties of the hydrated Co(III)_LS_-POR revealed a stable structure in an aqueous solution, thus suggesting the Co(III) ion to be in a low-spin state when bound to the porphyrin ring. Moreover, the structural and dynamical data were augmented by computing the free energy of water binding to the cobalt ions and the solvent-accessible surface area, which provide further information on thermochemical properties of the metal–water interaction and the hydrogen bonding potential of the porphyrin ring in these hydrated systems.

## 1 Introduction

Metal ions are known to be an integral part of the porphyrin molecule (POR) in nature, forming metalloporphyrin complexes that demonstrate diversified functions. For instance, metalloporphyrins are present in heme, cytochrome A, chlorophyll, etc.^1^ In addition, synthetic variants of metalloporphyrins were designed for specialized purposes considering the simple and straightforward accommodation of different metals in the core of the porphine ring, thus resulting in metalloporphyrins with enhanced functional diversity in numerous ways. Typical examples are photosensitizing properties in catalysis and dyes in photodynamic therapy (PDT),^2^ potential ionophore agents of chloride, fluoride, and nitrile, and biosensors for the detection of oxygen in organisms, as well as having a significant role in the conversion of solar energy.^3,4^ The incorporation of metal ions into porphyrins showed increased stabilization effects that improve their structural planarity in addition to enhancing thermodynamic as well as kinetic stability.^5−7^ The coordination of metal ions to porphyrins resulted in N-ligated porphyrin complexes after the deprotonation of one to two protons of the pyrrole ring NH moieties, thus resulting in metal–porphyrin complexes of variable structures displaying coordination geometries with a number of occupied coordination sites ranging from four to six.^8^

Apart from the most abundantly found natural metalloporphyrins in the form of heme and chlorophyll, their cobalt-containing counterparts were investigated in analogy to the core structure of vitamin B12, which has a fundamental role in the nervous system and acts as a cofactor in several metabolic processes. For instance, it is considered to be a crucial cofactor of the methionine synthase activity along with l-methylmalonyl coenzyme A mutase.^9^ However, the structural framework of vitamin B12 encapsulates a cobalt atom as the metal center in an aromatic tetrapyrrole macrocycle referred to as corrin, in which one of the methine bridges of the porphyrin structure is absent.^10−13^ Due to the existence of metal-bound porphyrins in nature incorporating specific chelating ligands, porphyrinoids have always attracted increased interest. In this regard, several studies have been reported that demonstrate the insertion of different metals including inter-alia iron, cobalt, copper, magnesium, manganese, and zinc in the porphyrin core structure with variable electronic configurations of the d-orbitals, resulting in distinctive magnetic, spectroscopic, and electrochemical properties.^14−16^

Besides, the chemistry of the cobalt metal itself demonstrates unique properties such as variable oxidation states ranging from −1 to +4 with Co(II) and Co(III) being the most common oxidation states forming different coordination complexes in a biological environment. In addition, Co^1+^ and Co^4+^ species exist as well, forming a variety of different complexes.^17^ Co(III) complexes show a d6 electronic configuration commonly having octahedral low-spin complexes with diamagnetic and inert properties or, alternatively, high-spin complexes with 2 or 4 unpaired electrons.^18^ In contrast, Co(II) with a d7 electronic configuration can form 4-, 5-, or 6-coordinated complexes, with paramagnetic and labile properties.^19,20^ In the past, aqueous Co(II) and Co(III) ions were investigated for the evaluation of their structural and dynamical properties, both showing octahedral coordination geometries in water using both classical and ab initio QM/MM MD methods.^21,22^ Another study proposed the oxygen-carrier capabilities of cobalt–porphyrin by binding of dioxygen in aqueous media, which was investigated by employing UV–Vis and electron paramagnetic resonance (EPR) spectroscopy.^23^ The catalytic properties of cobalt–porphyrin were investigated in the reduction of CO_2_ to CO in water by utilizing first principle calculations in conjunction with a dielectric continuum model for the treatment of water.^24^ Further, the water-soluble Co(III)–porphyrin was proposed to be a significant catalyst for the hydration of terminal alkyne moieties in chemical structures, thus signifying a particular role of the metalloporphyrin in catalyzing the conversion of alkyne to methyl-ketone.^25^ A similar study was conducted that classified homogeneous Co(III)–porphyrins as water oxidation catalysts (WOCs).^26^ The binding of different small volatile organic compounds with cobalt–porphyrin complexes specifying different multiplicities of the metal was investigated using DFT calculations for the design of colorimetric sensor arrays.^27^

More importantly, a detailed comparison of the chemistry of iron porphyrins and cobalt corrins has been reported by Kasper P. and Ulf Ryde, in which 4-, 5-, and 6-coordinated systems of variable oxidation states of the metal with axially coordinated imidazole and methyl ligands were analyzed for their geometry and electronic configuration properties using DFT calculations.^28^ Co-porphyrins with large side chain derivatives have also been synthesized and investigated using NMR in water and nonaqueous solution.^29^ Later, further derivatives were tested and then subjected to theoretical studies.^30^ Despite intense research that has been performed for the evaluation of the structural and dynamical properties of cobalt–porphyrins, a thorough comparison of the hydration properties of Co(II)–POR and Co(III)–POR was required, which could aid in further revealing the specific spin-states of cobalt in Co(III)–POR since ambiguity persists for the preferred spin-state of the metal bound to a porphine ligand. To answer this question, the current study was focused on aqueous Co(II)–POR (quartet) and Co(III)–POR considering both high (quintet) and low (singlet) spin-states of the metal ion in the latter case, which were investigated by employing an advanced ab initio quantum mechanical charge field molecular dynamics (QMCF MD) simulation protocol.^31,32^ The inclusion of the low-spin Co(III)–porphyrin complex (Co(III)_LS_–POR) in this study was also to examine the relative stability of Co(III)–POR in water to investigate whether it exists as a low- or high-spin complex.^28^

## 2 Methods

Similar to the other hybrid quantum mechanical/molecular mechanical (QM/MM) approaches, the ab initio quantum mechanical charge field molecular dynamics (QMCF MD) formalism was applied to a variety of molecular systems in which a system is partitioned into two main regions; the quantum mechanical (QM) region contains the atoms or molecules of significant chemical relevance and the molecular mechanical region includes the surrounding environment described by suitable force field (FF) methods.^33,34,31,32^ To study the aqueous complex systems, the QMCF MD adopted a multilevel description in which the QM region was further divided into an inner core zone and an outer solvation layer, which were appropriately applied for the investigation of hydrated systems which range from small ions to composite anions and coordination complexes.^35,36^ The QMCF MD formalism has demonstrated substantial improvement of the accuracy of results which was reportedly achieved with a modest increase of the computational effort of the quantum mechanical treatment related to the electrostatic embedding method and the employment of the population analysis. Moreover, if a sufficiently large QM region is applied, no solute–solvent potential functions except for the Coulombic interactions between atoms located in the inner core zone and MM region are required which are based on quantum mechanically derived partial charges thus enabling to study a wide range of systems that are difficult to represent by empirical or ab initio generated force fields.^32,31,36^ The QMCF MD approach was implemented by interfacing the formalism with a suitable quantum mechanical program, for instance the parallel version of the TURBOMOLE program was employed in the current study.^37^ Based on the successful applications of the Hartree–Fock (HF) level of theory in simulations of aqueous metalloporphyrins resulting in a very good compromise between the computation time and accuracy of the results, the same level of theory along with LANL2DZ plus ECP and the 6-31G** basis sets for Co and nonmetal atoms was employed.^45−48,38,39^

### Structural Evaluation

Structural properties of hydrated Co(II)–POR and Co(III)–POR, along with a water-bound low-spin Co(III)–POR complex, were obtained via radial distribution functions (RDFs),^40^ primarily the metal–O_w_ RDFs, which gave detailed insight into the water coordination to the Co(II) and Co(III) ions. In addition to the metal–water RDFs, the N–H RDFs were also evaluated for nitrogen atoms of the porphyrin ring to identify possible hydrogen bonding between the solute and the solvent. The binding of water to the metal ion of these metalloporphyrins was expected to result in complexes of a certain geometry, which was evaluated by angular distribution functions (ADFs) involving the coordinating nitrogen atoms of the porphyrin ring and oxygen atoms of water molecules, thus yielding angular attributes of the hydrated Co–porphyrins. The number of the coordinating water molecules to the metal ion and the nitrogen atoms of the solute was then confirmed by computing the associated coordination number distributions (CNDs). The aqueous environment confers biological molecules like porphyrin rings to adopt an enhanced conformational flexibility that could be essential to carry out their biological functions. Therefore, the flexibility of cobalt–porphyrins in solutions was probed with the help of dihedral and improper torsional distributions that were then augmented by the averaged root-mean-square fluctuations (RMSFs) for all atoms of the porphyrin ring, excluding hydrogen atoms. Besides the 1D description of the solvated systems with the help of individual radial and angular distribution functions, a detailed analysis based on spatial distribution functions (SDFs) was also carried out. Furthermore, angular-radial distribution functions (ARDs) based on combining both the radial and the angular components resulting in 3D density plots provided detailed information on the solvent distribution around the solutes using the porphyrin moiety as a plane of reference.^36^

### Evaluation of Dynamics

Ligand mean residence times (MRTs) were computed for the water molecules involved in the metal–water coordination as well as in hydrogen bonding with the nitrogen atoms, utilizing the direct method in which a time interval t* defines a “successful/sustainable” exchange process by the minimum duration of the ligand’s displacement from its original coordination shell and was chosen based on the displacement of a ligand from its original position as 0.0 and 0.5 ps, denoted as τ_0.0_ and τ_0.5_, respectively.^41^ The value of 0.5 ps for the time interval was set as the lower limit determined for a sustainable ligand exchange that corresponds to the average H-bond lifetime in water.^41−43^*R*_ex_ is defined as the ratio of the number of ligand exchanges registered for t* 0.0 ps, *N*_ex_^0.0^, and for more than 0.5 ps, *N*_ex_^0.5^.^41^
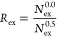
1

The integration of metal ions to the core of the porphyrin ring was reported to affect the rovibrational dynamics that were evaluated in terms of power spectra. Here, the spectra were computed for each solute via Fourier transform of the velocity autocorrelation functions, *C*(*t*) (VACFs),^44^
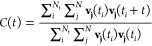
2using a correlation length of 2.0 ps with 5000 average time origins. Here *N* represents the number of particles and the number of time origins *t*_*i*_ is given as *N*_*t*_. The velocity components for particle *j* are indicated as *v*_*j*_.

### Characterization of Solute–Solvent Interactions

The direct binding of water to the metal ions of metalloporphyrins takes place due to coordinative unsaturation of the metal ions. Therefore, the computation of free energies of water binding to the metal ions was of great importance and quantified via the potential of mean force (PMF).^45−49^ The PMF also represented as *w*(*r*) in the literature can be expressed as
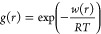
3where *R* and *T* denote the universal gas constant with a value of 8.314 J.K^–1^·mol^–1^ and the temperature in Kelvin, respectively. The radial separation *g*(*r*) is obtained from the corresponding RDFs.

The inherent relative hydrophilicity and hydrophobicity of the metalloporphyrins due to the presence of different atoms or groups of atoms were also evaluated in terms of the averaged solvent accessible surface area (SASA) using the program VMD,^50^ which was used to further characterize the relative H-bonding potential of these hydrated solutes.

### Simulation Protocol

The previously reported QMCF MD simulation of magnesium porphyrin in water served as the initial configuration for both systems, i.e., Co(II)–POR and Co(III)–POR in which Mg(II) was replaced by the respective cobalt ion.^45^ The initial structures of the high- and low-spin Co(III)–POR complexes were obtained in the same manner.^45^ The solutes were embedded in 2000 explicit water molecules forming a cubic simulation cell with a side length of 39.28 Å under periodic boundary conditions. The SPC/E water model (extended simple point charge model) was used to assign charges to the solvent particles located in the MM region.^51,52^ For the simulations, the isothermal–isobaric (NPT) ensemble was implemented using a time step of 2 fs and the velocity–Verlet algorithm was employed for the integration of the equations of motion.^53^ Bond lengths involving hydrogen atoms were constrained using the M-RATTLE algorithm which generally neglects the motion along bonding degrees of freedom of the atoms being constrained.^54,55^ In the NPT ensemble, temperature and pressure in the simulation were maintained at a constant value of 298.15 K and 1 atm using the Berendsen thermostat and manostat algorithms.^56^ All three simulation systems were partitioned according to specified parameters; the cobalt atom was assigned to be in the inner QM core zone set to a radius of 0.5 Å, and the solvation layer zone was set to a radius of 8.5 Å which constitute the overall QM region that was interfaced with the MM region via a smoothing region with a thickness of 0.2 Å to ensure frictionless transitions of crossing solvent molecules between these two regions.^32,31^ After an equilibration period of 2 ps, the systems were subjected to production MD for the sampling of simulation trajectories for a total of 10 ps per system.

## 3 Results and Discussion

### Structure

**Figure 1:**
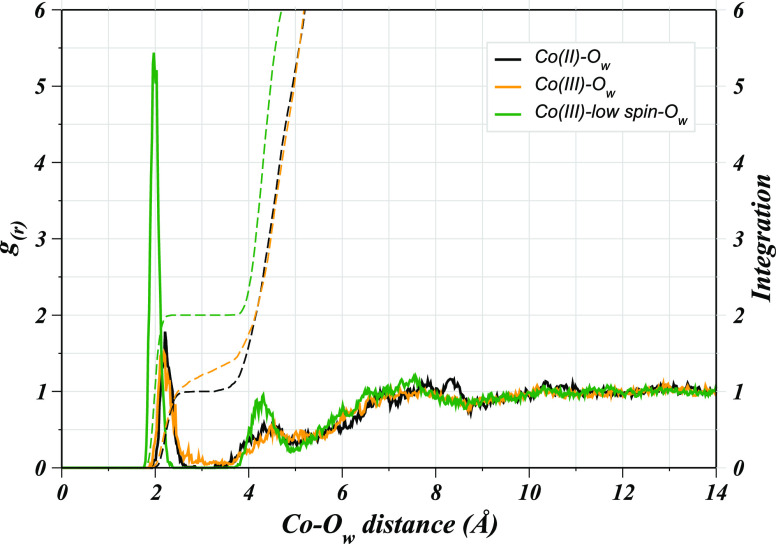
Radial distribution functions of water oxygens (O_w_) with respect to the cobalt ion in the three different cobalt porphyrins studied in this work.

For the evaluation of the structural properties of the hydrated solutes, the preliminary analysis based on the Co(II)–O_w_ and Co(III)–O_w_ RDFs already provides insight into the preferred spin-state of the two complexes. In the Co(III)–O_w_ RDF, a less intense first shell peak with a nonzero minimum is visible compared to the corresponding peak observed for the Co(II)–POR system, thus pointing toward an argument reported in the literature that the high-spin Co(III) complexes are considered less stable. However, complexes of the Co(II) ion could also be potentially present in the high-spin state.^57^ This argument was found to be reasonable based on the corresponding RDF for the low-spin Co(III)–POR (Co(III)_LS_–O_w_), which exhibited a sharp first shell peak with high intensity compared to the other two corresponding peaks (Figure 1). Detailed analysis of the Co(II)–O_w_ RDF revealed a well-structured first shell peak between 1.93 and 2.67 Å, which is well-separated from the second broader peak located between 3.27 and 5.03 Å connecting to the third peak found in the range from 5.43 to 8.75 Å, indicating ligand exchange events between the individual hydration shells. Integration of the Co(II)–O_w_ RDF up to the first shell minimum indicates an average of 1 axial water molecule directly coordinating to the metal ion. On the other hand, the corresponding peak in the Co(III)–O_w_ RDF differed strikingly, as the first shell peak had a nonzero minimum connecting to the broad second shell peak pointing toward an unstable hydrated Co(III)–POR complex. Nevertheless, the core of the analogous corrin rings was reported to be essentially suitable to accommodate the smaller Co(III) ion well for a stable complex compared to the porphyrin ring having a larger cavity.^58,57,59^ However, the ambiguity in the spin-states for the cobalt ions remained to be further explored which prompted for a detailed evaluation of the structure and dynamics of hydrated cobalt porphyrins. Therefore, the corresponding RDF for the low-spin Co(III)–POR complex (Co(III)_LS_–O_w_) was also obtained in comparison to the other two shown in Figure 1. The first shell peak in the Co(III)_LS_–O_w_ RDF appears at a shorter distance between 1.75 and 2.38 Å with approximately three times the intensity of the corresponding peak in the Co(II)–POR and Co(III)–POR cases, thus depicting a highly structured and rigid first hydration layer. Integration of the RDFs yielded an average of two axial water molecules to be present perpendicular to the porphyrin plane that did not exchange in the course of simulation time, as deduced from the zero minimum, following the broad second shell peak. Moreover, a peak splitting was observed indicating the possibility of hydrogen bonding between the metal-coordinated (axial) water and the second layer water molecules with the second broad peak appearing between 3.81 and 4.88 Å connecting to the third shell peak located in the range from 5.08 to 8.64 Å. The split peak could be a probability of axial water to have existed in two different forms either free and hydrogen bonded with the second shell water molecule thus corresponding to two different attributes of the first shell water molecules. The comparison of the Co(III)–O_w_ and Co(III)_LS_–O_w_ RDFs also demonstrated some contrast for the second and third shell peaks as the latter were intensified compared to the Co(III)–O_w_ case, and appeared at shorter distances. Based on the RDFs, the most probable metal–water coordination distance was found to be 2.20, 2.13, and 1.97 Å in cases of Co(II)–POR, Co(III)–POR, and Co(III)_LS_–POR, respectively.

**Figure 2:**
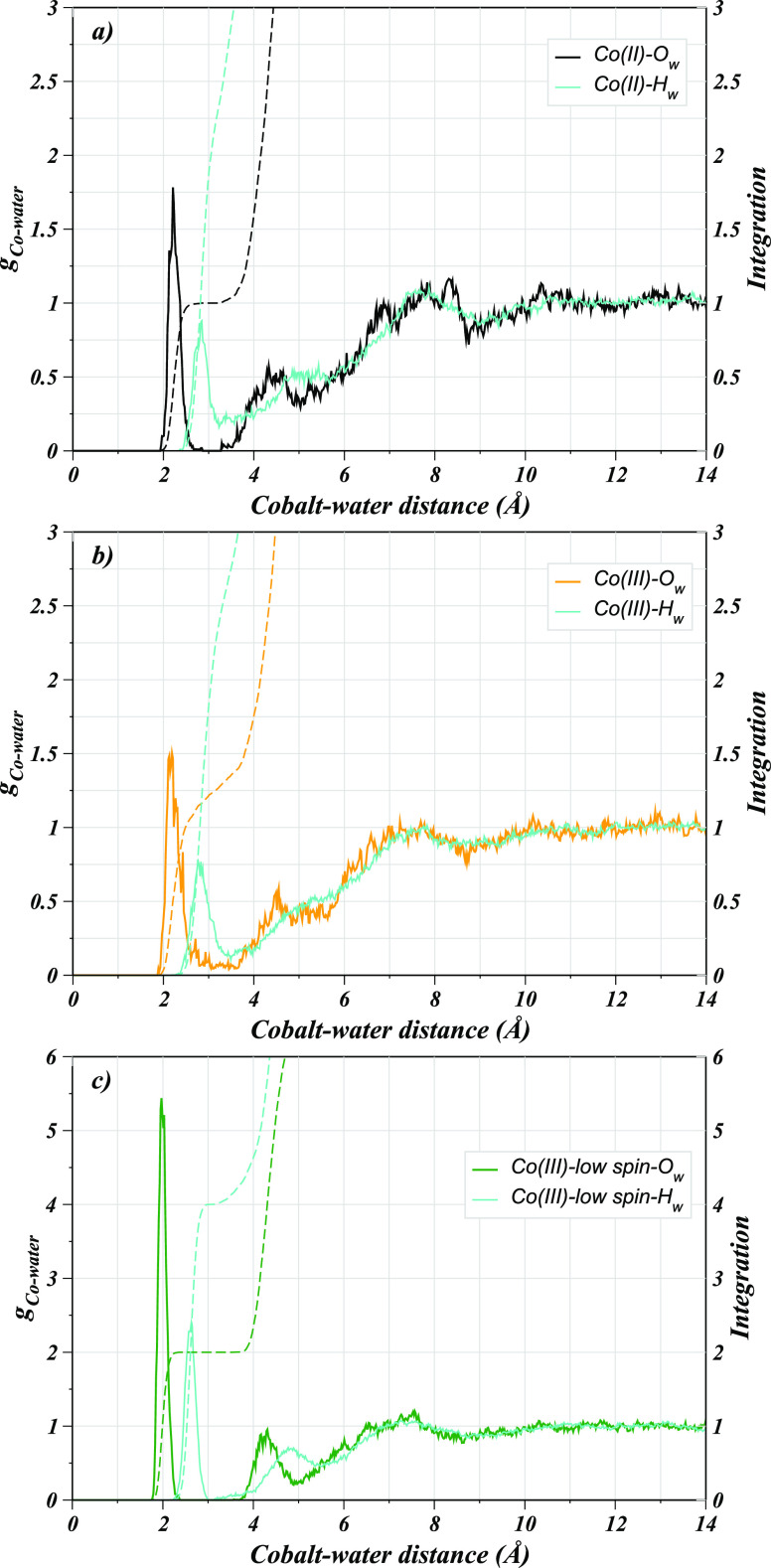
Radial distribution functions of water atoms with respect to the cobalt ion in aqueous (a) Co(II)–POR, (b) Co(III)–POR, and (c) Co(III)_LS_–POR.

Detailed insight into the hydration properties of these three metalloporphyrins was obtained via the computation of metal–hydrogen RDFs between the solute and solvent atoms in comparison to the metal–oxygen RDFs (Co^n+^–O_w_ and Co^*n*+^–H_w_, where *n* = 2, 3), as illustrated in Figure 2. The first peaks in the Co^*n*+^–H_w_ RDFs appear at larger distances and are less intense for all cases. The Co(II)–H_w_ RDF exhibits a pronounced first shell peak located between 2.35 and 3.21 Å and centered at 2.84 Å. The corresponding peak for the Co(III)–POR case is located between 2.19 and 3.48 Å with the average distance being 2.76 Å, showing also a nonzero minimum thus further envisaging interaction of metal-coordinated water molecule with those of the second solvation layer and possible water exchange between the individual hydration shells. In the Co(III)_LS_–H_w_ RDF, the first sharp peak is located between 2.19 and 2.97 Å with the most probable metal–water distance being 2.63 Å also displaying a nonzero minimum similar to the Co(III)_LS_–O_w_ RDF, which suggested a strong water-to-metal coordination, thus showing less possibility of hydrogen bonding with second shell water molecules. The comparison of the Co^*n*+^–O_w_ and Co^*n*+^–H_w_ RDFs provides detailed insight into the preferential localization of water molecules around the respective metal ions. It was also previously reported that the water molecules tend to interact with the porphyrin nitrogens via H-bonding which was later found to be diminished with the inclusion of the metal ions in the core of the porphyrin ring.^45−48^ A similar trend was observed in the case of the cobalt porphyrins as deduced from the N–H_w_ RDFs for all nitrogen atoms of the porphyrin ring (see Supporting Information).

As far as the geometry of these hydrated complexes was concerned, Co(II)–POR in aqueous solution adopts a 5-fold coordination geometry involving one water oxygen in addition to the four nitrogen atoms of the pyrrole rings coordinating to the metal ion. On the other hand, in the case of Co(III)–POR, a 5- or 6-fold coordination geometry can be observed, in which four nitrogen atoms were consistently bound to the metal. However, inconsistencies were observed due to varying coordination behavior of the ion with one or two axially coordinated water molecules. In the case of the Co(III)_LS_–POR, a distinct six-coordinated octahedral geometry was observed where two water molecules and four nitrogen atoms of the porphyrin ring were involved in the uninterrupted coordination to the metal. The findings based on the RDFs demonstrated varying structural attributes of these hydrated solutes which were linked to different electronic configurations of the metal ions thus driving the distinct metal–ligand interaction properties. The coordination behavior of these hydrated solutes is also in good agreement with that of the hydrated Co(II) and Co(III) ions reported using a similar simulation technique as water molecules are coordinating within ∼2.0 Å distance of the metal ions.^21,22^

**Figure 3:**
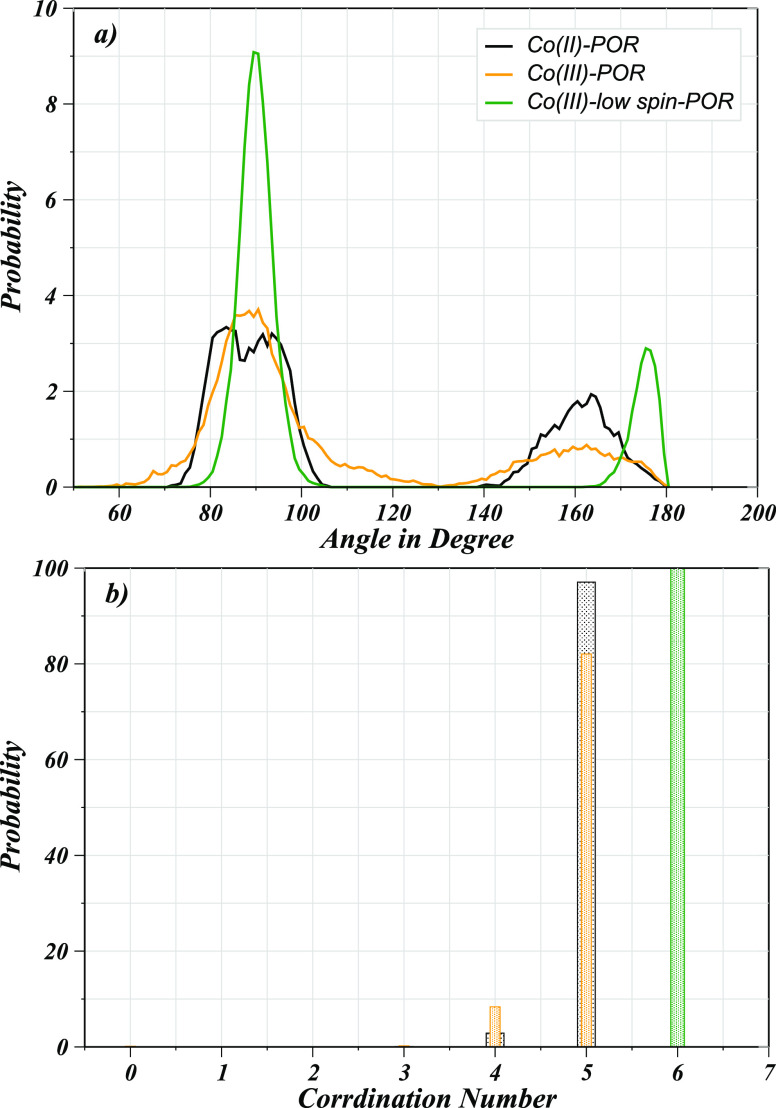
(a) Distribution of all registered X–Co–X (X = O, N) angles and (b) associated coordination number distribution within the first coordination layer for the cobalt ions bound to the porphyrin ring in aqueous solution obtained from the QMCF MD simulations.

**Figure 4:**
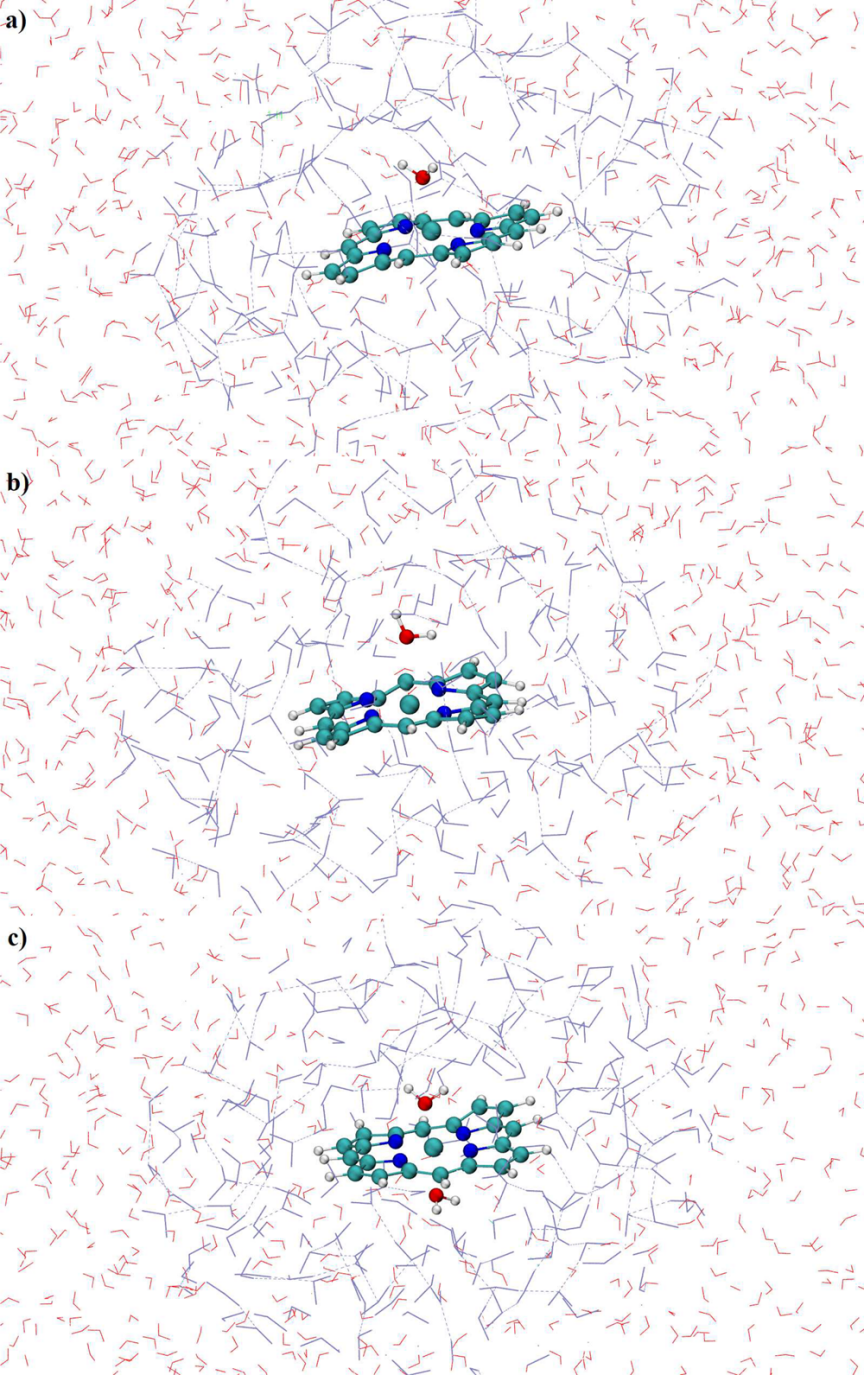
Snapshot of the (a) Co(II)–POR, (b) Co(III)–POR, and (c) Co(III)_LS_–POR in water taken from the simulation trajectories.

**Figure 5:**
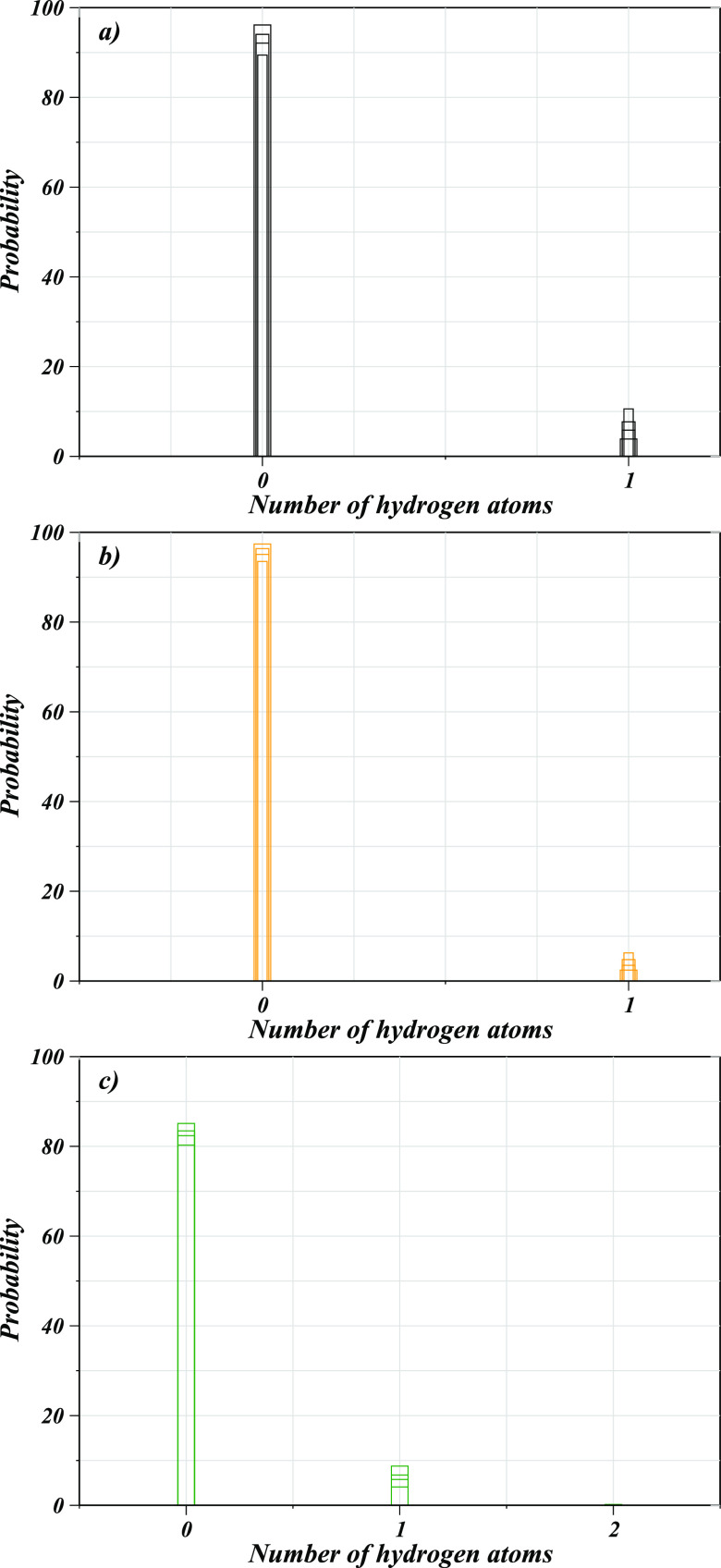
Coordination number distribution of H_w_ atoms involved in possible H-bonding with porphyrin nitrogens in (a) Co(II)–POR, (b) Co(III)–POR, and (c) Co(III)_LS_–POR, respectively.

To calculate the coordination geometry of these hydrated complexes, angular distribution functions (ADFs) and coordination number distributions (CNDs) were obtained as shown in Figure 3. The ADF provides a measure of the angle distribution of the metal atom coordinating to the atoms of the porphyrin ring and water molecules. The ADF plots depicted two profound peaks at ∼83° and ∼163° in the case of Co(II)–POR and Co(III)–POR, thus suggesting the distorted square pyramidal geometry. However, also a peak-splitting in the case of Co(II)–POR and Co(III)–POR was observed which hinted toward the possible existence of different coordination geometries, besides a 5-fold coordination structure since the computation of a definite geometry could not be achieved. In contrast, the Co(III)_LS_–POR complex demonstrated intense peaks at ∼90° and ∼177°, which are characteristic of an octahedral complex system^22^ (Figure 3a). In addition, the coordination number distribution (CND) depicted in Figure 3b quantified the coordination property in terms of number of the ligating atoms to the metal ions, for instance, a penta-coordination geometry can be deduced for the hydrated Co(II)–POR complex, whereas for the case of Co(III)–POR, the penta-coordinated geometry dominated a 4-fold coordinated geometry that occurred for short time intervals during the simulation. On the other hand, the Co(III)_LS_-POR case corroborated the ADF analysis indicating a hexa-coordinated geometry with zero possibility of other coordination geometries of the hydrated complex. Fluctuating coordination numbers and changes in the geometry occupancies are key characteristics of ligand exchange events observed within a quantum mechanical simulation. Moreover, the final complex structures were extracted from last configuration of the simulation trajectories displayed in Figure 4. The structures of the hydrated complexes showed a single water molecule coordinated to the metal ion in case of Co(II)-POR and Co(III)-POR, whereas Co(III)_LS_-POR demonstrated a binding of two water molecules in its first coordination layer thus showing a more stable structure as also deduced from the structural and dynamical properties evaluate so far. To obtain further access to the hydration properties of the second layer water molecules surrounding these solutes, a CND analysis was also performed for the ring nitrogens in connection to the hydrogen atoms of water (Figure 5a–c). The latter provides insights into the hydrogen bonding between the solutes and the solvent molecules, since the interaction of H_2_O to these solutes can be expected to stabilize the complexes in aqueous solution. Based on the CND analysis for the water hydrogens, all cases showed a very high probability distribution at zero thus demonstrating a very low probability of hydrogen bonding between the solutes and the solvent molecules. This was inferred as the strong preference of the metal ion toward the water oxygens thus repelling the respective hydrogen atoms.

**Figure 6:**
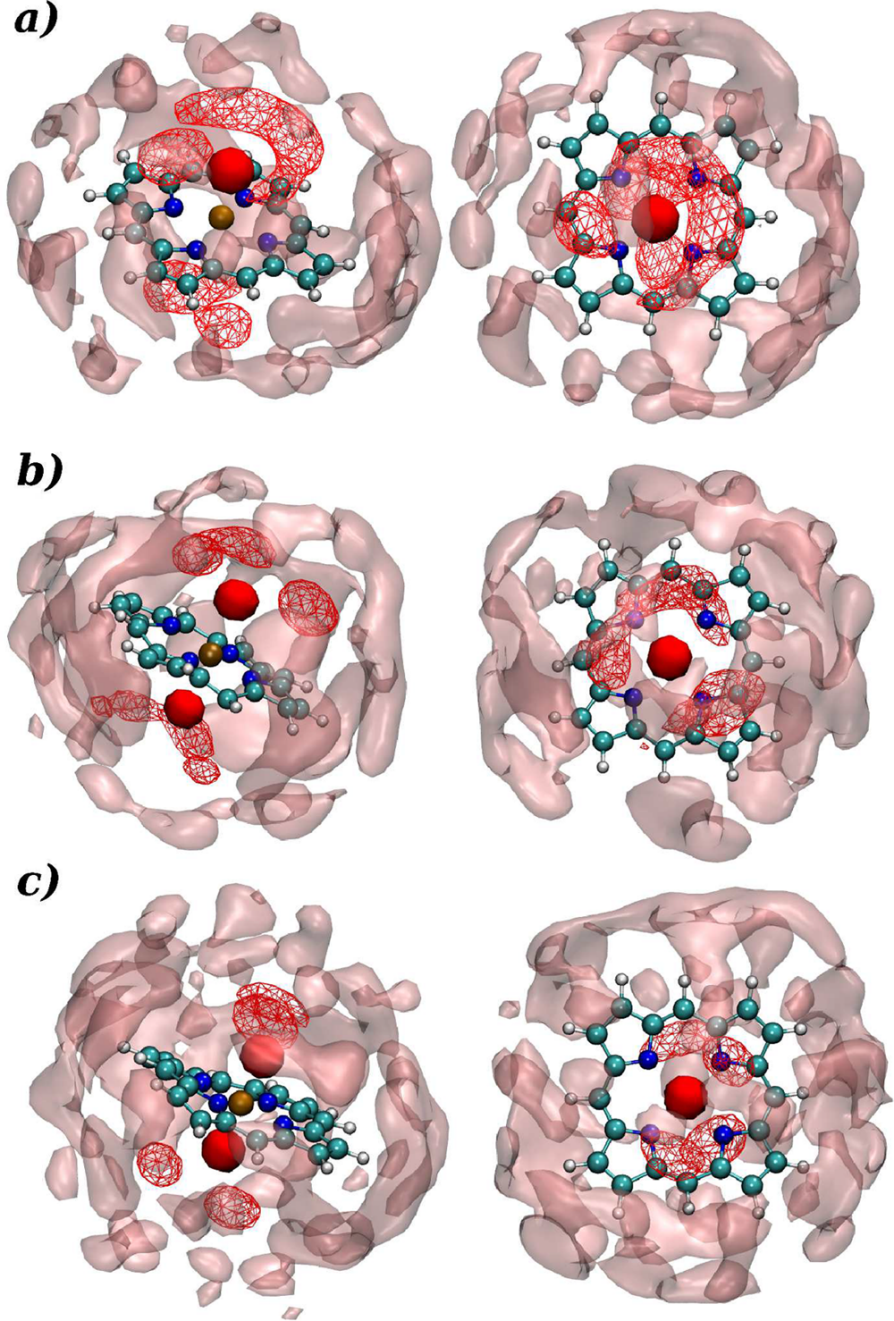
Isosurface representation of the spatial distribution functions depicting the oxygen density with respect to the (a) Co(II), (b) Co(III), and (c) Co(III)_LS_ ions of cobalt porphyrins in aqueous solution, (dark-red, solid: axial water molecules coordinating to metal ions; light-red, meshed: water molecules near solute nitrogens; translucent-red solid: hydration shell covering the entire solute).

To achieve an in-depth sketch of the water molecule’s orientation around the solutes, the 3D space defining the corresponding hydration shell of the metal was probed in terms of spatial distribution functions (SDF) derived from the information obtained through the RDF analysis.^36^Figure 6a depicts the SDF of the Co(II)–POR complex in an aqueous medium, in which binding of one axial water molecule was evident by the red solid sphere density. This is in line with the findings of the RDFs depicting the occurrence of a single axial-water molecule in the first hydration shell. The SDF plot obtained for hydrated Co(III)–POR displays a spatial density corresponding to two axial water molecules which thereby give rise to the idea of having six-coordinated species contrary to the other findings based on RDF, ADF, and CND (Figure 6b). However, SDF only provides an averaged density plot and since the possibility of ligand exchange remains as initially inferred, the depiction of two axial water molecules in the complex could also be the result of ligand exchange occurring on either side of the porphyrin plane. In the case of the Co(III)_LS_–POR depicted in Figure 6c, the ligand density corresponding to the axially coordinated water molecules correlates well with the results of the RDF, ADF, and CND analyses pointing toward a purely octahedral geometry of the complex. The wireframe depictions in the SDFs also highlight the second hydration layer occupying the spaces in the proximity of solutes’ nitrogens and the light-red outer surface represention corresponded to the third hydration layer which covered the entire solutes including axially bound as well as the second shell water molecules. The formation of the large third hydration layers resulted due to the hydrophobic nonpolar atoms at the periphery of the porphyrin ring that repelled the water molecules farther away thus forming a full hydration layer around the complexes. Since the metal ions display a preference for attracting water oxygens instead of hydrogen atoms, a reduced possibility of establishing H-bonding with the ring nitrogens is observed. Nevertheless, for the further appraisal of the bit contrasting CND data for these hydrated complexes, three-dimensional analysis was required for the evaluation of water density distribution in the vicinity of the complexes. Therefore, a closer inspection in three-dimensional space was required to further explore the solvent distribution surrounding these nonspherical systems.

**Figure 7:**
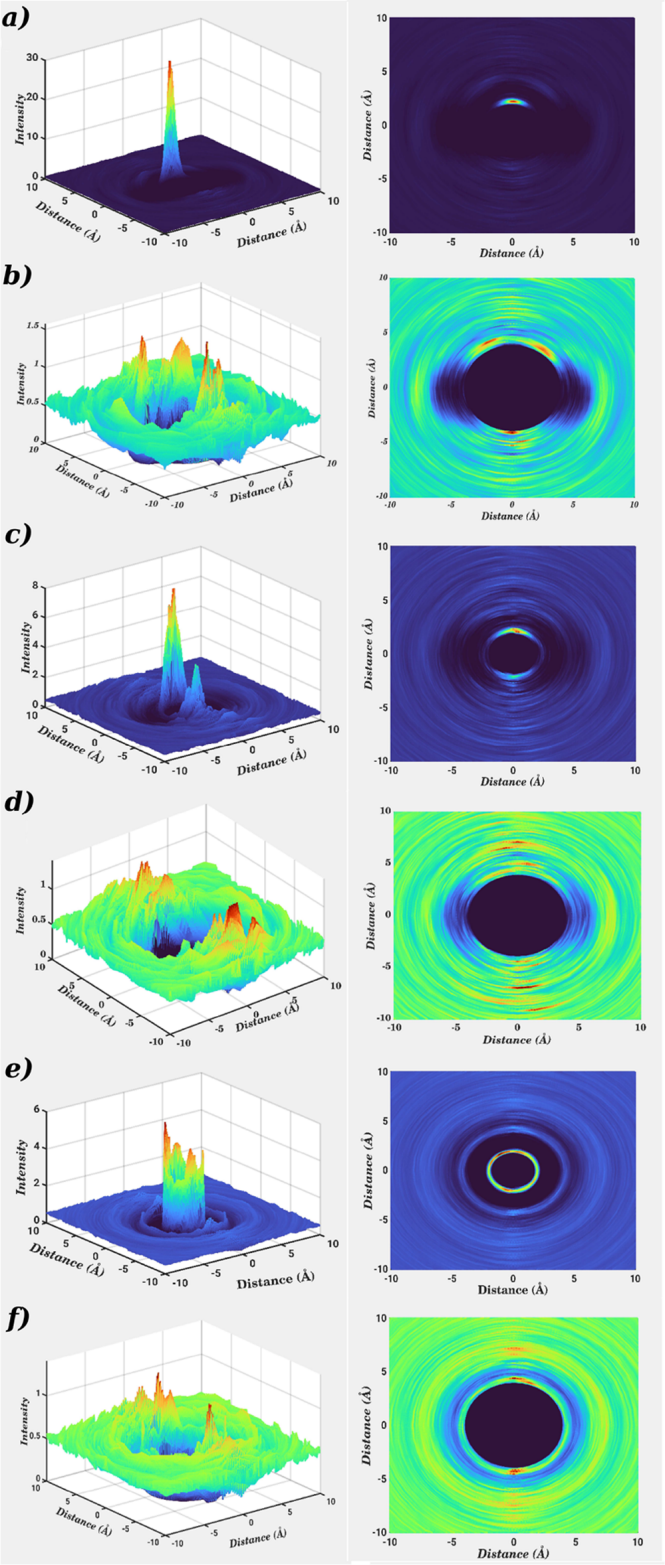
Angular radial distribution contour plots of the oxygen density with respect to (a) Co(II), (c) Co(III), and (e) Co(III)_LS_ ions of cobalt porphyrins in aqueous solution. The density contribution of axial water molecules has been omitted for clarity in order to assess the distribution of water molecules in the proximity of the porphyrin nitrogen of (b) Co(II)–POR, (d) Co(III)–POR, and (f) Co(III)_LS_–POR (color scheme for respresenting density of water molecules: red, high; yellow, medium; and light blue, low intensity).

To access the hydration pattern of the investigated Co–porphyrins, angular-radial distribution (ARD) plots were generated which inherently combine angular and radial components with respect to the positions of the metal ion and the four nitrogen atoms of the porphyrin ring defining the molecular plane. Based on the metal–O distance and the angle to the normal vector of the porphyrin plane, the respective density profiles of water oxygens can be determined. Figure 7a–f depicts the ARD plots providing detailed information on the hydration patterns of the three solutes. In the case of Co(II)–POR, a single sharp and intense peak appeared representing the presence of an axially coordinated water molecule in the proximity of the cobalt ion (Figure 7a). The corresponding plot demonstrated an asymmetric hydration toward Co(III)–POR since two density peaks of unequal intensities were observed corroborating the RDF, ADF, and SDF analysis (Figure 7c). However, in the case of hydrated Co(III)_LS_–POR, an enhanced symmetry in the peak distribution and intensity was observed which corresponds to the first hydration layer demonstrating that the two axial positions of the metal ion were occupied by water molecules (Figure 7e). To further explore the solvent density in the second hydration layer, a second set of ARD plots was generated by subtracting the density of the metal-coordinated water molecules, thereby achieving a better visualization of the water distribution on either face of the porphyrin ring plane without the influence of the first shell water density. Figure 7b,d,f displays the ARD plots for the second hydration shell to investigate potential hydrogen bonding between the solutes and the solvent molecules; for the case of Co(II)–POR, a relatively high water density was observed compared to the Co(III)–POR and Co(III)_LS_–POR cases, which demonstrated that the increasing electro-positivity of the metal affected the hydrogen bonding propensity. On the other hand, the ARD plots for Co(III)–POR exhibited a scattered distribution thus signifying ligand exchanges between the second and third hydration layer.

**Figure 8:**
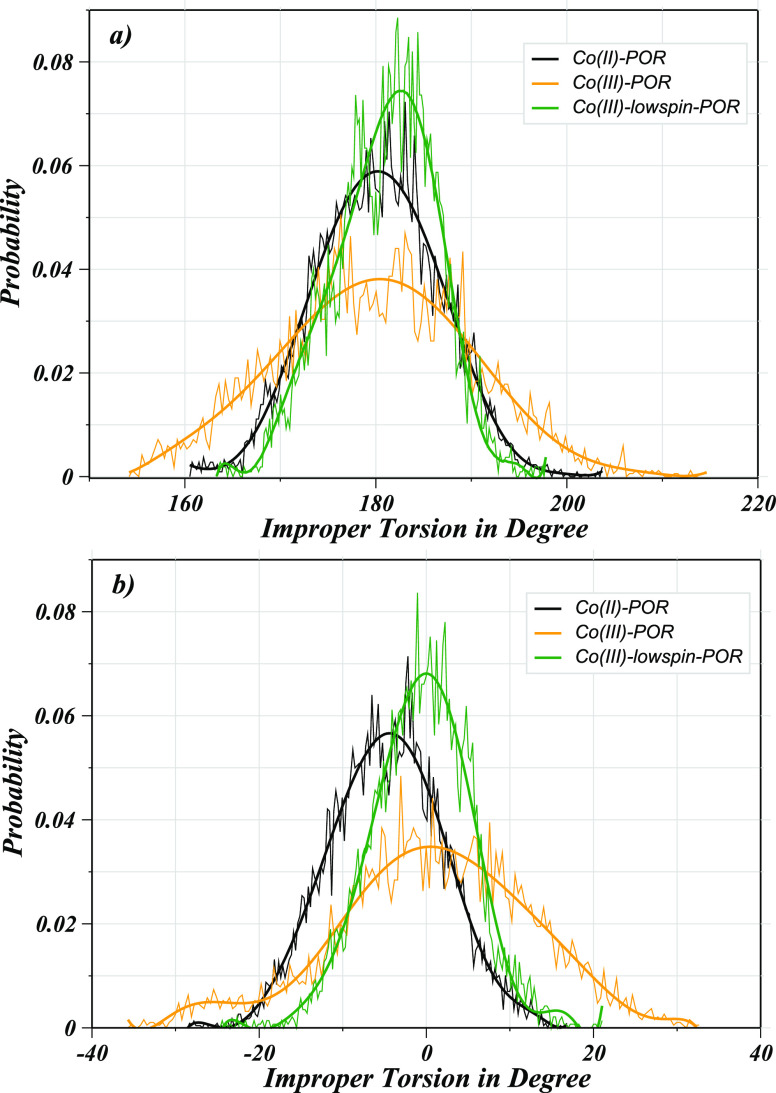
Averaged torsional distribution functions formed between (a) the nitrogen atoms of the porphyrin systems (N–N–N–N) as well as (b) the cobalt ion and the pyrrole rings (Co–C–C–N) obtained obtained for the cobalt porphyrins via QMCF MD simulations (the thick, solid line represented a running average over the averged torsional distributions, i.e., one and four in the cases of the N–N–N–N and Co–C–C–N dihedral angles, respectively.

**Figure 9:**
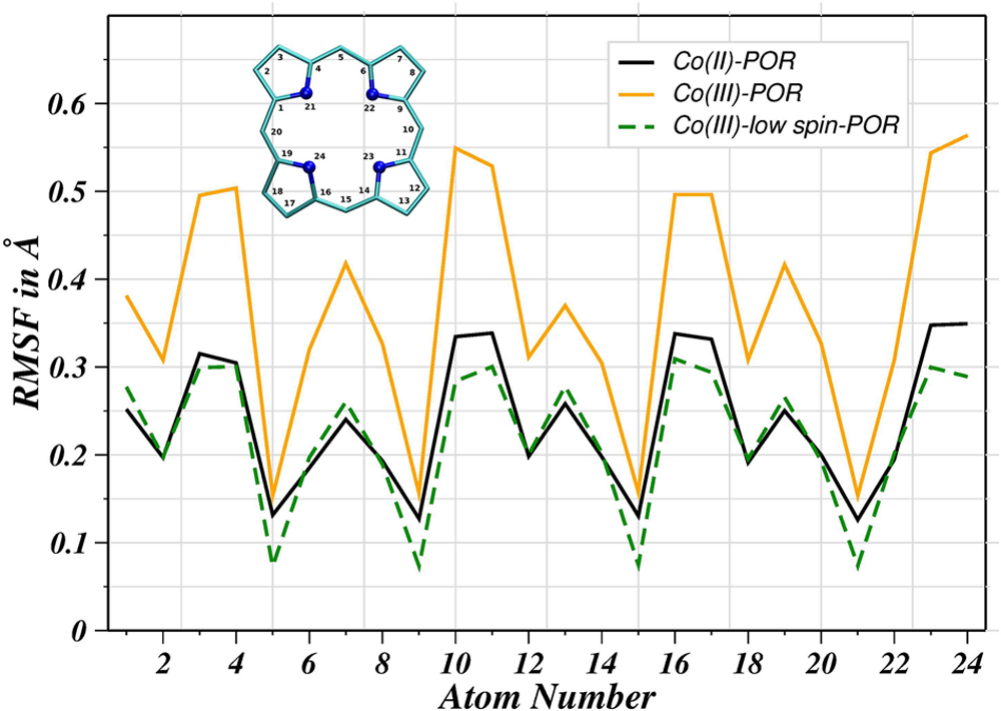
Atomic root-mean-square fluctuations of heavy atoms of the porphyrin ring of cobalt porphyrins after alignment of the simulation trajectories.

The hydration behavior of these metalloporphyrins was associated with the conformational freedom of the porphyrin ring that is known to be reduced after the insertion of the metal ions in the ring core.^45^ Nevertheless, aqueous metalloporphyrins were reported to have some residual degrees of freedom which were also explored for the three considered solutes involving the nitrogen atoms of the pyrrole rings in terms of distribution functions for dihedral and improper torsional angles as displayed in Figure 8. The improper torsional terms provide a measure of the macrocyclic ring’s perturbation in response to metal incorporation, which bears a specific electronic structure since the metal-free porphyrin ring displays an elevated structural flexibility in aqueous media.^45^ Based on the distribution functions, these three solutes were found to show variations in the torsional distributions for instance narrow peaks were observed for both dihedral and improper torsional angles in the case of Co(III)_LS_–POR compared to other two complexes. This finding implies that the low-spin Co(III) ion formed a more rigid complex which underwent a distortion in the geometry of the complex upon coordination by water molecules. This distortion was attributed to the strong coordination between the cobalt ion and axial water molecules thus resulting in the displacement of the metal from the plane of porphine ring.^45^ Besides, the evaluation of the comformational flexibility was further characterized via a root-mean-square fluctuation (RMSF) analysis carried out for all the heavy atoms excluding the metal ion in all solutes after aligning the trajectories. Figure 9 illustrates the RMSF profiles of these solutes which overall display patterns. However, concerning the literature on the metal-free porphyrin system,^45,47^ the accommodation of cobalt ions in the macrocyclic ring structure was presumed to perturb the overall flexibility of the porphyrin structural framework. Here out of the three cases, the aqueous Co(III)–POR complex exhibits a pronounced increase in atomic fluctuations compared to the other two cases and this behavior correlates with the RDF findings demonstrating a highly unnatural fragile first hydration shell of the complex as well as the possible occurrence of the first shell ligand exchange. On the other hand, the corresponding complex in a low-spin electronic configuration was found to form a stable configuration as deduced from the low atomic fluctuations, thus suggesting the formation of a rigid first hydration layer. This stable Co(III)_LS_–POR complex in aqueous solution was a clear indication of nature’s preference for the Co(III) ion to be in a low-spin state, as reported in the literature for the existence of the cobalt corrin complex.^60,28^

### Dynamics

**Figure 10:**
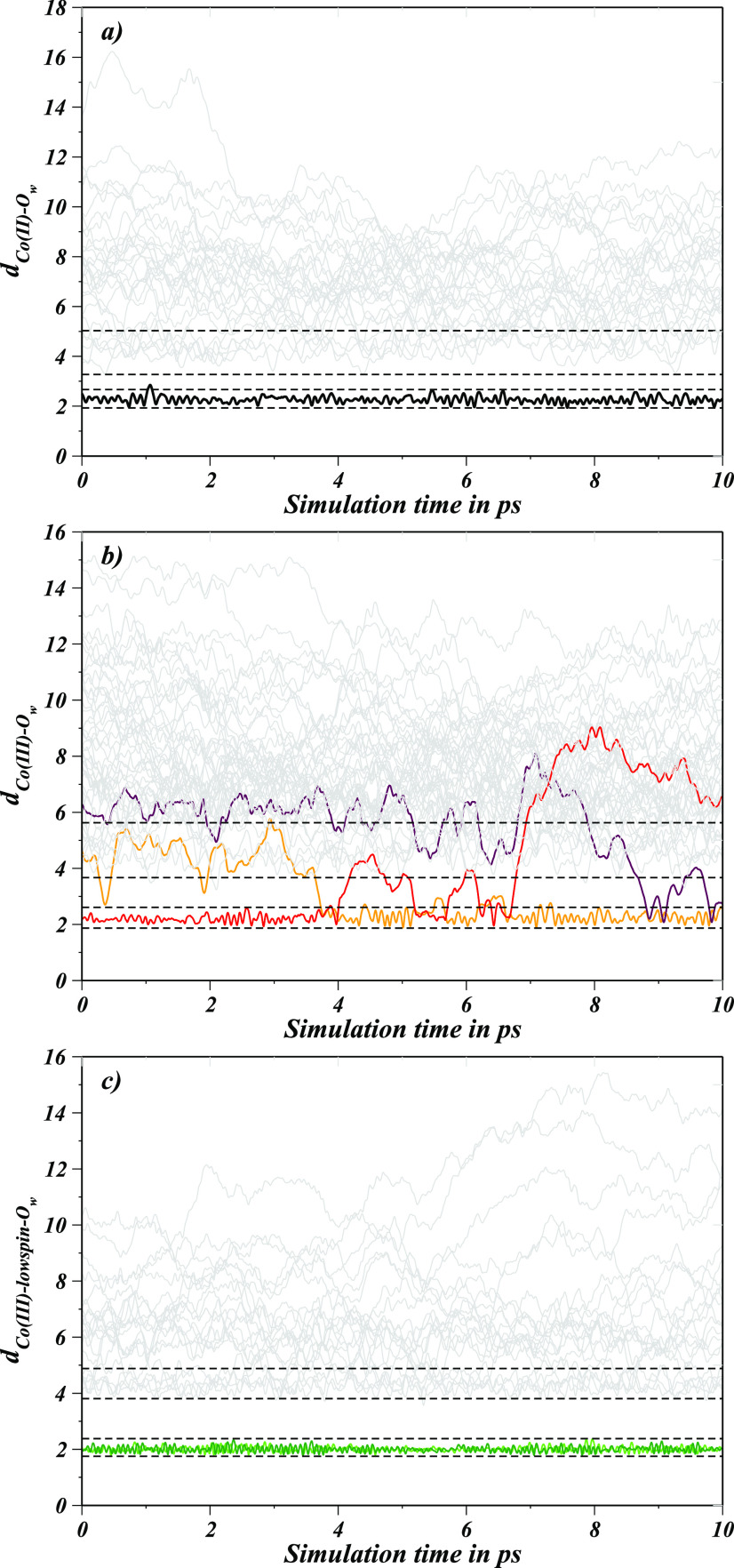
Metal–oxygen distance plots for (a) Co(II)–POR, (b) Co(III)–POR, and (c) Co(III)_LS_–POR registered along the simulations. Horizontal dashed lines represent boundaries of hydration layers identified from the pair distribution functions, selected distance plots involved in ligand exchange are highlighted in color.

The hydrogen bonding potential of the N–H_w_ bonds was further evaluated and inspected between solutes and solvents via ligand mean residence times (τ_0.5_) for all three hydrated systems in comparison to that in the metal-free porphyrin (4.7 ps).^45^ The calculated MRT values for the N–H_w_ bond in the hydrated Co(II)–POR, Co(III)–POR, and Co(III)_LS_–POR were calculated as 0.6, 0.2, and 0.4 ps, respectively (Table 1). The reduction in MRT of the N–H_w_ bond in these hydrated metalloporphyrins was inferred as the metal insertion to the porphyrin ring core perturbed the relative orientation of water atoms causing the water hydrogens to be away from the ring plane.^45−48^ Besides the MRTs, other relevant data, including the respective sustainability coefficient, which is the reciprocal of the number of successful water exchanges between the first and second hydration layers, were further determined via distance plots showing the possible water exchanges between different hydration layers.^41^ There was no exchange of water molecules between the first and the second hydration layers observed for Co(II)–POR and Co(III)_LS_–POR. On the other hand, the Co(III)–POR case demonstrated water exchanges between the first and the second hydration layers which were attributed to the very fragile hydration layers of the high-spin cobalt–porphyrin complex. To obtain further insight into the water exchange dynamics for all three hydrated cobalt–porphyrin complexes, metal–oxygen distance plots were generated, as depicted in Figure 10a–c, which exhibited no water exchange between the first and the second hydration layer of Co(II)–POR. However, frequent water exchanges took place between the second and the third hydration layer as well as with the bulk solvent. Contrary, Co(III)–POR displayed water exchange events from 4 to 10 ps, where three water molecules were observed to leave their respective hydration layers thus showing at least two successful exchange events along with many exchange reactions between the third hydration layer and the bulk. In the case of the Co(III)_LS_–POR, no water exchange occurred between the first and second hydration layers which also validated the initial assessment based on the Co(III)_LS_–O_w_ RDF.

**Table 1:** Dynamical Data for Water Molecules Forming Possible H-Bonding with Nitrogen Atoms of the Porphyrin Ring in Co(II)–POR, Co(III)–POR, and Co(III)_LS_–POR in Comparison to the Metal-Free Porphyrin

H-bond (system)	CN	*N*_ex_^0.5^	*N*_ex_^0.0^	τ_0.5_ (ps)
N-Hw (metal-free porphyrin)^45^	∼1	∼1	264	4.7
N-Hw (Co(II)–POR)	0.1		48	0.6
N-Hw (Co(III)–POR)	0.1		33	0.2
N-Hw (Co(III)_LS_–POR)	0.1		110	0.4

**Figure 11:**
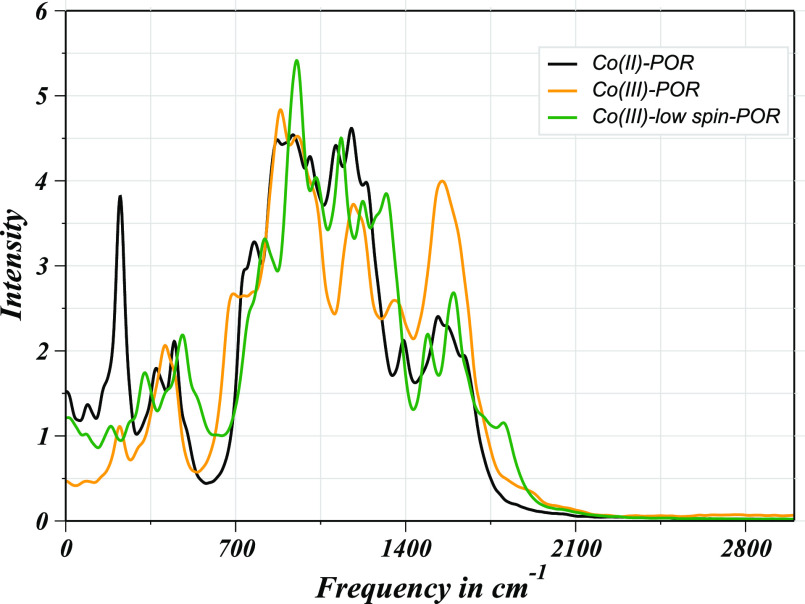
Vibrational power spectra of aqueous cobalt porphyrins.

Fourier-transformed velocity autocorrelation functions were utilized to determine vibrational power spectra of all the investigated Co–POR complexes to represent their rovibrational modes by taking all atoms excluding hydrogen atoms into account due to the consideration of constrained H atoms during the QMCF MD simulation (Figure 11).^54,55^ The computation of power spectra for the porphyrin atoms reveals a frequency shift that occurred due to the incorporation of cobalt ions into the core of the porphyrin ring, as these hydrated systems demonstrated drastic differences in their spectral patterns relative to the metal-free porphyrin as reported earlier using the same simulation method.^45^ The highest band in the case of Co(II)–POR, Co(III)–POR, and Co(III)_LS_–POR, was located at approximately 1178, 885, and 952 cm^–1^, respectively, which were the obvious representation of the spectral shift in all these cases compared to the case of metal-free porphyrin (approximately 940 cm^–1^).^61,45^ Apart from these values, less variation in the wave numbers and band intensities were observed for the hydrated Co(II)–POR and Co(III)_LS_–POR complexes, thus attributing to the existence of stable complexes in aqueous solution. Minor differences in the wave numbers can be attributed to the cobalt ions having different electronic structures which consequently changed the vibrational modes of the respective porphyrin framework. In the case of Co(II)–POR, an additional intense band also appeared near ∼225 cm^–1^, which could be associated with the strong Co(II)–O_w_ vibration correlating the spectral data reported for the aqueous Co(II) ion studies via the QM/MM MD study.^21^ On the other hand, the spectral pattern of the Co(III)–POR case demonstrated strongly contrasting characteristics compared to the other two cases, which is linked to the presence of a very fragile hydration complex of the solute in water discussed above. The distinct vibrational modes of these hydrated solutes were also correlating with the RMSF data thus supporting the argument of structural perturbation of the porphyrin ring by the cobalt insertion.

### Solute–Solvent Interactions

**Figure 12:**
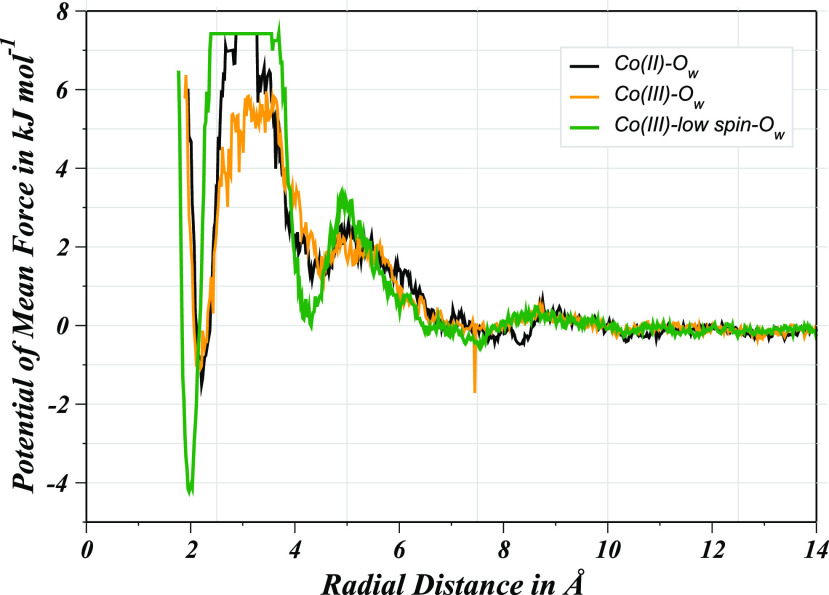
Potential of mean force of solvent molecules derived from the *g*(*r*) profiles for aqueous Co(II)–POR, Co(III)–POR, and Co(III)_LS_–POR.

The water binding to the metal ion was further analyzed in terms of the respective binding energy which was significant to complement the description of the structural and dynamical properties.^45−47,49^ For the computation of the water-binding affinity to the cobalt ion in aqueous Co(II)–POR, Co(III)–POR, and Co(III)_LS_–POR complexes, the potential of mean force (PMF) was utilized for the quantification of water-to-metal coordination in different hydration layers as a function of *g*(*r*). Figure 12 illustrates the PMF profiles of the respective solute–solvent interaction with the energetics of the first hydration layer coordination which were as followed, Co(II)–POR: −1.54 kJ mol^–1^, Co(III)–POR: −1.10 kJ mol^–1^, and Co(III)_LS_–POR: −4.23 kJ mol^–1^. The negative PMF values suggested a stronger ion–water coordination, and in this case, the hydrated Co(III)_LS_–POR possessed the most negative binding energy due to the enhanced stability of the hydrated solute, thus demonstrating that the low-spin state of Co(III) ion is also preferred from the perspective of the ion–ligand interaction even.

The hydrogen bonding potential of cobalt porphyrins with water was further estimated through the evaluation of relative hydrophobicity/hydrophilicity of the respective metalloporphyrins with reference to the previously reported metal-free porphyrin in aqueous solution.^45^ It was also reported that the inherent residual hydrophobicity and hydrophilicity of the porphyrin ring were reported to be perturbed by the incorporation of a metal ion in the ring core. Similarly, the time-averaged SASA was computed for the hydrated Co(II)–POR, Co(III)–POR, and Co(III)_LS_–POR complexes along with the metal-free porphyrin (Table 2). The SASA data demonstrated the enhanced hydrophobicity of the porphyrin ring due to inclusion of the cobalt ion thereby causing the hydrogen bonding potential of these hydrated solutes with water molecules to be diminished. The largest value for hydrophobic surface area was obtained for the hydrated Co(II)–POR followed by Co(III)–POR thus confirming that the insertion of Co(II) ion to the porphyrin core significantly reduced the hydrogen bonding potential of the porphyrin ring, despite the relative hydrophilicity of these two systems being close. On the other hand, the Co(III)_LS_–POR case showed the lowest hydrophobic and hydrophilic surface area. The trend in the hydrophilic surface area in these hydrated solutes consequently justified the preferential orientation of water atoms toward these solutes, as evidenced by the case of the hydrated Co(III)_LS_–POR, which possessed at least two axially coordinated water molecules.

**Table 2:** Time-Averaged Solvent Accessible Surface Area in nm^2^^a^

porphyrins	hydrophobic	hydrophilic	total
metal-free porphyrin^45^	4.58	0.29	4.87
Co(II)–POR	4.98	0.09	5.06
Co(III)–POR	4.84	0.09	4.93
Co(III)_LS_–POR	4.79	0.06	4.85

a Hydrophobic and hydrophilic surfaces are decomposed to show their individual contributions.

## 4 Conclusion

The current study successfully implemented the QMCF MD formalism for the investigation of hydration properties of three cobalt–porphyrin systems. In this work, cobalt was studied in different electronic configurations due to its significant natural occurrence in corrin complexes of biological systems, which prompted us to investigate cobalt in the parent porphyrin structures in two oxidation states, Co(II) and Co(III), along with a definite low-spin Co(III) since it was suggested to be also present in low spin complexes. Based on the simulation results, the Co(III)_LS_–POR in aqueous solution was found to be structurally and dynamically stable compared to its high-spin analog. The structural and dynamical properties of these cobalt complexes obtained from the QMCF MD simulations imparted a significant body of knowledge on the natural existence of the cobalt ion in specific electronic configurations to form stable complex with porphyrins and/or corrins. This could be made possible only by incorporating high-level quantum mechanics in the molecular dynamics framework thus providing detailed information on the hydration properties of the cobalt porphyrin complexes. Besides the more-commonly evaluated data, free energies of water binding to the metal ion and the inherent hydrophobicity/hydrophilicity based on the solvent-accessible surface area provided further insight into the characteristics of the hydrated Co(III)–POR complex in the high- and low-spin state in comparison to the Co(II)–POR complex in aqueous solution. Furthermore, the findings based on the simulation results paved the way to evaluate other metalloporphyrins including their analogous complexes like cobalt corrins that naturally exist, thus also enabling the simulation method to be a platform for the investigation of complex metal-containing biological molecules embedded in biological environment.

## Supporting Information Available

Radial distribution function of water hydrogens with respect to the nitrogen atoms of the porphyrin ring of (a) Co(II)–POR, (b) Co(III)–POR, and (c) Co(III)_LS_–-POR showing a very low probability of N–H bond in all cases (PDF)
