# Supplementary material for: Deciphering Structural and Dynamical Properties of Hydrated Cobalt Porphyrins via Ab Initio Quantum Mechanical Charge Field Molecular Dynamics Simulation
Source: J Phys Chem B. 2023 May 23;127(22):5072–83. doi: 10.1021/acs.jpcb.3c00837 (PMC10258806; doi:10.1021/acs.jpcb.3c00837)
Supplement: Supplementary file 1 — jp3c00837_si_001.pdf [file jp3c00837_si_001.pdf]

Deciphering Structural and Dynamical Properties of Hydrated Cobalt Porphyrins *via* Ab initio Quantum Mechanical Charge Field Molecular Dynamics Simulation

Sehrish Jamal, Zobia Naz, Syed Tarique Moin*

Third World Center for Science and Technology, H.E.J. Research Institute of Chemistry
International Center for Chemical and Biological Sciences
University of Karachi, Karachi-75270, Pakistan
Tel.: +92-21-99261774
Fax: +92-21-34819018

Thomas S. Hofer†

Theoretical Chemistry Division
Institute of General, Inorganic and Theoretical Chemistry
University of Innsbruck, Innrain 80-82, A-6020 Innsbruck, Austria
Tel.: +43-512-507-57102
Fax: +43-512-507–57199

Dated: May 8, 2023

*Electronic address: tarique.syed@iccs.edu
†Electronic address: T.Hofer@uibk.ac.at; Corresponding author

Supplementary Information

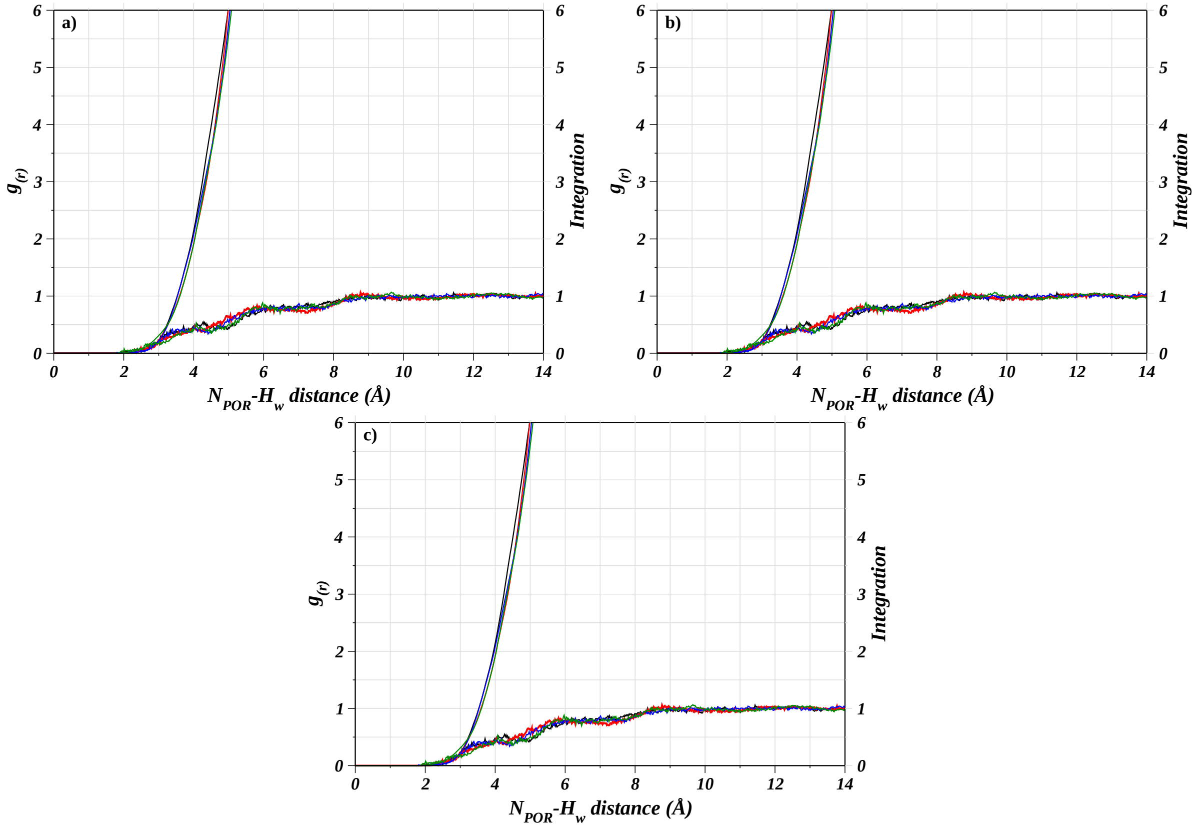

RDFs of hydrogen (Hw) atoms of water, with respect to the nitrogen atoms of the porphyrin ring in (a) Co(II)-POR, (b) Co(III)-POR and (c) $Co(III)_{LS}$-POR.
